# Using the Canadian Longitudinal Study on Aging to examine the association between tooth loss and hip bone mineral density

**DOI:** 10.3389/fdmed.2026.1602549

**Published:** 2026-02-23

**Authors:** Jenalyn L. Yumol, Diane P. Dupont, Wendy E. Ward

**Affiliations:** 1 Department of Kinesiology, Brock University, St. Catharines, ON, Canada; 2 Centre for Bone and Muscle Health, Brock University, St. Catharines, ON, Canada; 3 Department of Economics, Brock University, St. Catharines, ON, Canada

**Keywords:** bone mineral density, CLSA, diet, dual x-ray absorptiometry, oral health, osteoporosis

## Abstract

**Introduction:**

Given the care gap for osteoporosis among Canadians, in part due to barriers limiting access to a diagnostic bone density scan, this study investigated the association between number of teeth and bone mineral density (BMD) to help identify individuals at-risk of osteoporosis. The purpose of this study was to determine if an individual's number of teeth was associated with hip BMD in Canadian men and women aged 50–85 years using cross-sectional data from the Canadian Longitudinal Study on Aging (CLSA).

**Methods:**

Eligible participants (*n* = 20,629) underwent an in-home questionnaire to collect responses including, number of teeth (< or ≥ 20 teeth), demographic variables (age, sex, ethnicity, body mass index, income) and lifestyle habits (current smoking status, physical activity level, diet). Hip BMD was measured using dual x-ray absorptiometry. The association between number of teeth (< or ≥ 20 teeth) and hip BMD was evaluated using a multiple linear regression with a *post-hoc* analysis by T-score categorization (normal bone mass, low bone mass, osteoporosis).

**Results:**

Number of teeth was not significantly associated with an individual's hip BMD when adjusted for major risk factors associated with osteoporosis and oral health (age, sex, ethnicity, body mass index, current smoking status, income, physical activity level, and diet). However, varying results were observed when hip BMD was categorized by T-score. In participants with low bone mass, having fewer than 20 teeth was associated with lower hip BMD (*P* = 0.040). Whereas, having fewer than 20 teeth was associated with higher hip BMD in participants with normal bone mass (*P* < 0.001). No significant association between number of teeth and hip BMD was found in the osteoporosis group (*P* = 0.360).

**Conclusion:**

There was a lack of association between number of teeth and hip BMD in the combined study sample. The divergent associations seen for normal and low bone mass require further investigation. Whether including other indicators of oral health - in addition to number of teeth - can strengthen the ability to predict hip BMD with or without considering hip BMD status is an area of future study.

## 1 Introduction

With the inevitable decline in bone mass during adulthood, proactive efforts to identify intervention strategies to support bone health and reduce the risks of osteoporosis and related fragility fractures are needed. However, the development of osteoporosis may be unnoticed until a fracture occurs. Fractures lead to increased risk of excess mortality and decreased quality of life (1), with a higher risk of mortality demonstrated following a hip fracture compared with a non-hip fracture (2). Measurement of bone mineral density (BMD) by dual x-ray absorptiometry (DXA) at the hip is the clinical gold standard for assessing bone health and estimating an individual's risk of fracture (3). Yet, a DXA test is elective, not included in universal healthcare costs, and is therefore not regularly performed. A care gap has been reported by the Public Health Agency of Canada, identifying that “of those who had an osteoporosis-related fracture, less than 20% received an osteoporosis diagnosis, underwent a BMD test or received a prescription for an osteoporosis-related medication within one year of the fracture” (4). Determining surrogate measures of bone health, such as number of teeth, provides an opportunity to identify those at-risk of osteoporosis and fracture.

Osteoporosis and periodontal disease link whole body bone health and oral health as they share several risk factors (age, sex, ethnicity, smoking status, physical activity and diet) (5–7), and both are also associated with inflammation-induced bone resorption (8, 9). The highest prevalence of these bone diseases is among older adults (10, 11). In particular, one of the major reasons for tooth loss with aging is the onset of periodontal disease, in which there is damage to the jawbone and the surrounding soft tissue that supports teeth (12). The relationship between periodontal disease and osteoporosis has been reported in systematic reviews and meta-analyses investigating the association between lower BMD and poorer periodontal disease outcomes (13, 14). The standard reporting of a combination of periodontal disease outcomes have been previously proposed, including periodontal bone resorption, pocket probing depth, clinical attachment loss, and bleeding upon probing (15). However, with less frequent annual dental visits among older adults, compared with younger adults, and over 1 in 5 Canadians reporting not going to a dental professional because of cost-related barriers (16), using these periodontal disease outcomes may be challenging for at-risk individuals. Self-assessment of the number of teeth as a surrogate measure for bone health is an observable and meaningful outcome that is accessible. The importance of improving oral health, including tooth retention, to promote overall health has been recognized as a global public health issue that should be integrated into the mainstream health system to promote healthy aging (17–20). This vision has been brought to the spotlight by the World Health Organization through the “Global strategy and action plan on oral health 2023–2030” (18) as well as Japan's “8020” campaign to promote the retention of 20 teeth by age 80 years to achieve good health, including greater BMD (19), and higher quality of life among the elderly (20). A recent scoping review suggested that older individuals with fewer than 20 teeth were likely to experience poorer health (21). Number of teeth is an objective measure of tooth loss and has been previously quantified continuously or dichotomously using a cutoff value for functional dentition. A widely used benchmark for functional dentition is the presence of 20 or more functional teeth as it is generally accepted to be the minimum number of teeth needed to support basic oral functions such as chewing (22). Thus, functional dentition plays a major role in maintaining overall health, including bone health, by providing the ability to consume a healthy, balanced diet. Diet is a modifiable risk factor influencing BMD and the retention of teeth. Dairy foods are known to support BMD and retention of teeth by providing a rich source of calcium and Canada's cow's milk is fortified with vitamin D, another key nutrient that supports bone and tooth health (3). Moreover, dietary patterns that are higher in fruits, vegetables, nuts and legumes, whole grains and protein-rich foods (poultry, fish) have also been positively associated with bone health (23). For individuals with 20 teeth or fewer, it is difficult to consume some of these foods, thus influencing dietary choices to accommodate challenges with chewing (24), and in turn increasing the risk for malnutrition.

Krall et al. (1996) was among the first to report on the relationship between tooth loss and BMD at multiple skeletal sites (whole body, hip, spine). Healthy, White, postmenopausal women participated in 3 intervention studies in which calcium or vitamin D supplementation was provided (25). Regardless of the level of supplementation with calcium and vitamin D, reductions in whole body, hip, and spine BMD were associated with an increased risk of tooth loss (25). Since then, studies have been conducted in the USA (25–30), Japan (31–33), Poland (34, 35), Turkey (36), Lativa (37), Greece (38, 39), Finland (40), and United Kingdom (41, 42), but not Canada, to examine the relationship between tooth loss and BMD in men and women. While many studies have shown an association between lower BMD and greater tooth loss, others have found no association.

The overall aim of this cross-sectional study was to determine if a dichotomous measure of an individual's number of teeth (< or ≥ 20 teeth) was associated with hip BMD in Canadian men and women aged 50–85 years using data from the Canadian Longitudinal Study on Aging (CLSA). A significant association could provide support for using tooth loss to identify individuals at risk for low bone mass. Counting the number of teeth is relatively simple and does not require specialized equipment or technology compared with the clinical measurement of BMD using DXA.

## 2 Methods

The CLSA is a national study aimed at understanding the multifaceted changes that accompany aging to support strategies for improving the health and quality of life of Canadians (43). Using the CLSA research platform, this current study undertook a cross-sectional analysis of follow-up 1 data from the comprehensive cohort (Follow-up 1 Comprehensive Dataset - Version 5.0). Full details about participant recruitment and eligibility criteria have been previously reported (43, 44). In brief, eligible participants were English or French speaking, had no cognitive impairment at baseline and were willing to participate in both in-home interviews as well as data collection at the data collection site (43, 44). Demographic (age, sex, ethnicity, BMI, income) and lifestyle variables (smoking, alcohol, physical activity, diet) were integrated to provide insight into additional covariates that contribute to loss of bone mass.

All research activities of the CLSA abided by the ethical conduct requirements of the Canadian Institutes of Health Research (Tri-Council Policy Statement: Ethical Conduct for Research Involving Humans, TCPS). This present study was conducted in accordance with research based on secondary use of data, as approved by the Brock University Health Science Research Ethics Board (File #22-239).

### 2.1 Primary outcome: hip BMD

There were 11 data collection sites (located in Victoria, BC; Vancouver, BC; Surrey, BC; Calgary, AB; Winnipeg, MB; Hamilton, ON; Ottawa, ON; Montréal, QC; Sherbrooke, QC; Halifax, NS; or St. John's, NL) where a measurement of hip BMD was performed using a Hologic Discovery A DXA machine (https://www.clsa-elcv.ca/doc/524). The region of interest for the total hip was delineated superior to the base of the lesser trochanter. Participants were excluded if they were pregnant, unable to stand without assistance, involved in a Nuclear Medicine study within the previous 2 days, had a prosthetic, or history of a break or fracture (https://www.clsa-elcv.ca/doc/1236).

### 2.2 Primary predictor variable: a dichotomous variable indicating fewer than 20 teeth

Based on the data collection site questionnaire on oral health (https://www.clsa-elcv.ca/doc/1236), a dichotomous variable was created to identify participants with 20 or more natural teeth vs. fewer than 20 natural teeth. Individuals that responded, “don't know/no answer” were characterized as missing and not included in the analysis.

### 2.3 Covariates: age, sex, ethnicity, BMI, smoking, income, physical activity, diet

Previous findings have shown that age, sex, ethnicity, BMI, current smoking status, income, physical activity level [Physical Activity Scale for the Elderly (PASE) score], and diet are all contributing factors that may modulate bone health (3, 45). Demographic data and lifestyle data were collected by interviewees during the in-home interviews (https://www.clsa-elcv.ca/doc/1047).

Current smoking status was derived using “At the present time, do you smoke cigarettes daily, occasionally or not at all?” and/or “Do you currently use any other types of tobacco products?”, in which a yes response to both or either question indicated that the individual was a current smoker. Recoded data for current smoking status was used to do the analysis.

The CLSA questionnaire collected personal and household income data with the use of categories ($0 to $10,000; $10,000 to $35,000; $35,000 to $75,000; $75,000 to $125,000; $125,000 to $175,000 and above $175,000). To facilitate ease of coefficient interpretation, these categories were recoded to create a new variable that represented the midpoint within each income range as the personal or household income used in the analysis. Since survey subjects were interviewed over a four-year period (2015–2018), this means that an income of $100,000 (equivalent to category 3) for someone interviewed in 2015 does not represent the same purchasing power as the same nominal income ($100,000) identified for a person in 2018. During that period, the average level of inflation (increase in general level of prices, including food) was around 2%. So, a subject with a $100,000 income in 2018 would be able to purchase less food than one with the same $100,000 at 2015 prices. Instead of using so-called nominal income (in prices of the current year), economists use real income (adjusted to represent real purchasing power of a given basket of goods in what is called the reference year). Statistics Canada uses the year 2002 as the reference year and calculates a consumer price index to measure the rate of inflation from both the reference year to any other year, as well as the rate of inflation between two or more subsequent years. Total personal income and household income were standardized to the reference year 2002 (2002 = 100) relative to the participant's province of residence and interview date (month and year) to account for inflation over the interview period (2015–2018) (46). The inclusion of both measures of income was intended to capture the variation in an individual's budget from all available sources (personal and household support). To account for households with multiple persons, the household income can provide information of additional financial support.

Physical activity was quantified using a PASE score to capture the total frequency, duration and intensity of typical physical activities of older adults (leisure activities, household activities, as well as work and volunteer activities) performed over a weekly period (47). The PASE score ranged from 0 to 793 and a higher score was associated with a greater physical activity level (47).

For diet, the CLSA Nutrition: Short Diet Questionnaire was used to evaluate the usual intake of foods over the previous 12 months (48). The food questions were categorized as total daily intake of one of ten food categories: fruits, vegetables, nuts, legumes, fish, dairy, meats, whole grains, calcium fortified foods and beverages, and fibre (Table 1).

**Table 1:** Categorization of foods according to the specified questions from the CLSA nutrition: short diet questionnaire.

Food category	CLSA Nutrition: Short Diet Questionnaire
Fruits	“How often do you usually eat fruit (fresh, frozen, canned)?”
Vegetables	“How often do you usually eat: green salad (lettuce, with or without other ingredients); carrots (fresh, frozen, canned, eaten on their own or with other food, cooked or raw); other vegetables (except carrots, potatoes or salad)?”
Nuts	“How often do you usually eat nuts, seeds and peanut butter?”
Legumes	“How often do you usually eat legumes: beans, peas, lentils?”
Fish	“How often do you usually eat salmon, trout, sardines, herring, tuna, mackerel (fresh, frozen or canned)?”
Dairy	“How often do you usually eat: all low-fat cheeses; all regular cheeses; yogurt (low-fat); yogurt (regular)?” “How often do you usually drink: calcium-fortified milk (35% more calcium); whole milk 3.25% m.f.; 2%, 1%, skim milk?”
Meats	“How often do you usually eat: beef, pork (ground, hamburgers, roast beef, steak, cubed..); other meats (veal, lamb, game?) (ground, hamburgers, roast, steak, cubed, ..); chicken, turkey?”
Whole grains	“How often do you usually eat whole wheat breads, bran breads, multigrain breads, ryebreads (sliced, crusty, hamburger bun, hot dog bun, bagel, pita, ..)?”
Calcium fortified foods and beverages	“How often do you usually eat calcium-fortified foods (soy pudding, ..)?” “How often do you usually drink: calcium-fortified juices; other calcium-fortified beverages (soy drink, ..)?”
Fibre	“How often do you usually eat high fibre breakfast cereals (All Bran, 100% Bran, Bran Flakes, muesli..)?”

### 2.4 Statistical analyses

All statistical analyses were performed using IBM SPSS Statistics software (version 29.0.1.0) and assuming a significance of *p*-value < 0.05. The association between hip BMD (dependent variable) and the dichotomous variable whether a respondent had 20 or more teeth or fewer than 20 teeth (predictor variable) was evaluated using multiple linear regression. Data were analyzed using three models incorporating variables describing major risk or moderating factors associated with osteoporosis and periodontal disease: number of teeth and age (Model 1); number of teeth, age, sex, ethnicity, BMI, and current smoking status (Model 2); and number of teeth, age, sex, ethnicity, BMI, current smoking status, income, physical activity level, and diet (Model 3). Multicollinearity among covariates was assessed using a variance inflation factor (VIF) threshold of 5 to indicate a strong correlation. *Post hoc* subgroup analysis on each of the models was also done after using the T-score classification to group respondents into one of three groups: normal bone mass, low bone mass, and osteoporosis (49, 50). Data are presented as mean ± standard error.

## 3 Results

### 3.1 Study population

**Figure 1:**
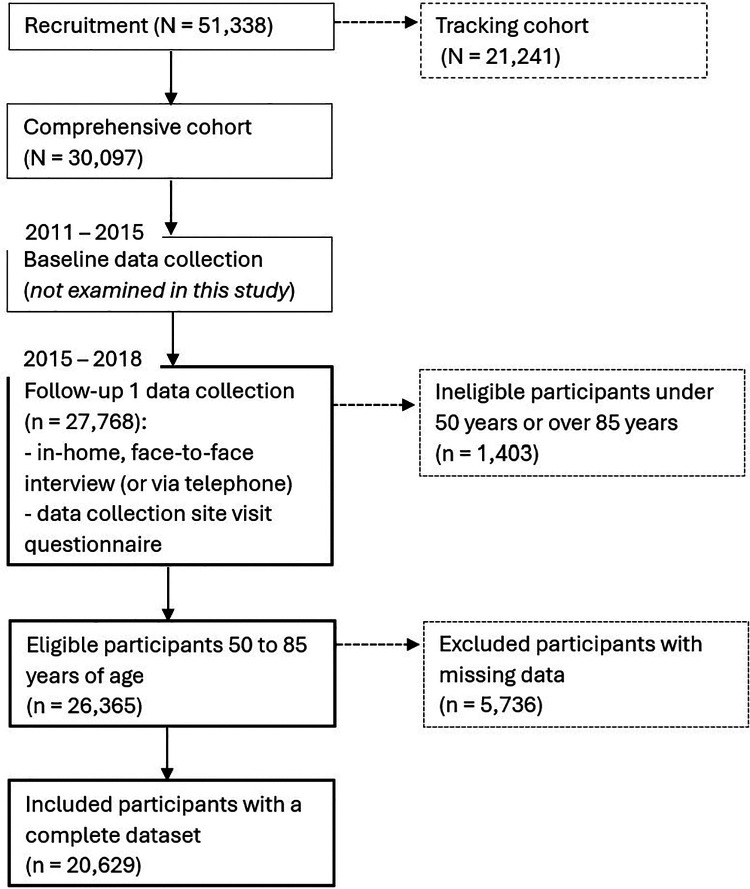
Participant flow diagram.

The study population was inclusive of community dwelling Canadian men and women aged 50–85 with a complete dataset (*n* = 20,629; 49.3% females; mean age = 64.92 ± 0.06 years; Figure 1). Descriptive summaries of the demographic and lifestyle variables included in the models are described in Table 2 for the combined sample and the subgroups classified by T-score for BMD (normal bone mass, low bone mass, osteoporosis). There was no correlation between any of the predictor variables within the models (VIF < 5) both for the combined sample and subgroups. For the combined sample, participants with missing data (*n* = 5,736) were excluded as the missing data was not completely random (Little's missing completely at random test, *P* < 0.05) – those that were less likely to report a response for hip BMD and/or number of original teeth were older, had lower income, and a lower level of physical activity.

**Table 2:** Descriptive summaries of covariates examined within this study (*n* = 20,629).

Variable [reference category]	Males		Females	
	≥ 20 teeth	< 20 teeth	≥ 20 teeth	< 20 teeth
**Sample size**				
All participants	8,512	2,003	8,234	1,979
Normal bone mass	6,233	1,315	3,326	647
Low bone mass	2,101	616	4,174	1,103
Osteoporosis	137	60	693	223
**Hip BMD, g/cm^2^**				
All participants	0.81 (0.001)	0.80 (0.003)	0.72 (0.001)	0.70 (0.003)
Normal bone mass	0.86 (0.001)	0.87 (0.003)	0.83 (0.001)	0.83 (0.004)
Low bone mass	0.68 (0.001)	0.67 (0.002)	0.66 (0.001)	0.66 (0.001)
Osteoporosis	0.54 (0.003)	0.54 (0.01)	0.54 (0.001)	0.54 (0.003)
**Age, years**				
All participants	63.97 (0.10)	70.84 (0.19)	63.13 (0.10)	70.45 (0.19)
Normal bone mass	63.14 (0.11)	70.11 (0.24)	60.66 (0.14)	68.18 (0.33)
Low bone mass	66.06 (0.20)	72.17 (0.33)	64.40 (0.13)	71.30 (0.25)
Osteoporosis	69.50 (0.79)	73.62 (1.17)	67.37 (0.34)	72.85 (0.51)
**White ethnicity**				
All participants	96.5%	97.4%	97.4%	98.3%
Normal bone mass	96.66%	97.64%	97.14%	97.37%
Low bone mass	96.00%	97.08%	97.82%	99.09%
Osteoporosis	96.35%	95.00%	95.96%	97.31%
**BMI, kg/m^2^**				
All participants	28.26 (0.05)	28.39 (0.11)	27.78 (0.07)	28.59 (0.14)
Normal bone mass	28.92 (0.06)	29.40 (0.13)	29.85 (0.11)	31.14 (0.26)
Low bone mass	26.57 (0.09)	26.79 (0.17)	26.70 (0.08)	27.66 (0.16)
Osteoporosis	24.47 (0.33)	22.84 (0.48)	24.42 (0.18)	25.71 (0.36)
**Current smoker**				
All participants	9.0%%	14.2%	6.5%	11.3%
Normal bone mass	9.03%	11.86%	6.49%	10.97%
Low bone mass	8.81%	18.83%	6.30%	11.60%
Osteoporosis	10.95%	18.33%	8.37%	10.76%
**Total personal income^a^**				
All participants	54,814.78 (342.25)	37,456.50 (560.98)	39,023.36 (293.02)	26,144.24 (428.15)
Normal bone mass	56,317.00 (401.25)	38,931 (705.54)	40,539.65 (483.09)	26,470.80 (761.56)
Low bone mass	50,880.43 (666.48)	35,198.07 (952.40)	38,457 (398.36)	26,350.16 (578.05)
Osteoporosis	48,080.89 (2,693.57)	30,355.71 (3,103.04)	35,050.83 (910.71)	24,538.17 (1,136.91)
**Total household income^a^**				
All participants	74,022.82 (375.38)	50,601.42 (663.73)	64,179.02 (383.20)	39,814.99 (619.19)
Normal bone mass	76,005.92 (158.77)	52,281.36 (829.21)	67,882.37 (612.81)	41,343.47 (1,130.11)
Low bone mass	68,998.95 (733.70)	48,212.97 (1,153.06)	62,853.99 (525.88)	39,863.64 (809.97)
Osteoporosis	60,812.20 (3,164.60)	40,675.95 (3,385.17)	54,422.41 (1,264.25)	35,181.69 (1,764.69)
**PASE score**				
All participants	154.82 (0.82)	127.20 (1.55)	136.91 (0.77)	109.97 (1.34)
Normal bone mass	158.77 (0.97)	129.61 (1.90)	143.17 (1.27)	114.94 (2.40)
Low bone mass	144.77 (1.55)	124.06 (2.78)	134.14 (1.03)	108.37 (1.79)
Osteoporosis	125.40 (6.29)	100.86 (8.39)	124.14 (2.34)	103.95 (3.57)
**Total daily intake of fruits, times/day**				
All participants	1.15 (0.01)	1.03 (0.02)	1.47 (0.01)	1.34 (0.02)
Normal bone mass	1.15 (0.01)	1.03 (0.02)	1.47 (0.02)	1.35 (0.04)
Low bone mass	1.15 (0.02)	1.03 (0.03)	1.46 (0.02)	1.34 (0.03)
Osteoporosis	1.22 (0.08)	0.99 (0.08)	1.47 (0.04)	1.32 (0.06)
**Total daily intake of vegetables, times/day**				
All participants	1.64 (0.01)	1.47 (0.02)	2.09 (0.01)	1.82 (0.02)
Normal bone mass	1.63 (0.01)	1.46 (0.02)	2.08 (0.02)	1.81 (0.04)
Low bone mass	1.66 (0.02)	1.50 (0.04)	2.10 (0.02)	1.82 (0.03)
Osteoporosis	1.71 (0.09)	1.39 (0.12)	2.10 (0.04)	1.86 (0.07)
**Total daily intake of legumes, times/day**				
All participants	0.30 (0.003)	0.26 (0.01)	0.26 (0.003)	0.20 (0.01)
Normal bone mass	0.29 (0.004)	0.26 (0.008)	0.25 (0.005)	0.20 (0.01)
Low bone mass	0.30 (0.01)	0.26 (0.01)	0.26 (0.004)	0.20 (0.01)
Osteoporosis	0.35 (0.03)	0.29 (0.05)	0.28 (0.01)	0.22 (0.02)
**Total daily intake of nuts, times/day**				
All participants	0.60 (0.01)	0.52 (0.01)	0.63 (0.01)	0.55 (0.01)
Normal bone mass	0.59 (0.01)	0.53 (0.01)	0.62 (0.01)	0.56 (0.02)
Low bone mass	0.63 (0.01)	0.50 (0.02)	0.63 (0.01)	0.54 (0.01)
Osteoporosis	0.57 (0.04)	0.57 (0.07)	0.70 (0.02)	0.60 (0.06)
**Total daily intake of fish, times/day**				
All participants	0.17 (0.002)	0.17 (0.004)	0.19 (0.002)	0.18 (0.004)
Normal bone mass	0.17 (0.002)	0.17 (0.004)	0.18 (0.003)	0.18 (0.01)
Low bone mass	0.18 (0.005)	0.16 (0.01)	0.19 (0.003)	0.18 (0.005)
Osteoporosis	0.17 (0.01)	0.19 (0.03)	0.22 (0.01)	0.20 (0.01)
**Total daily intake of dairy, times/day**				
All participants	1.63 (0.01)	1.63 (0.02)	1.74 (0.01)	1.73 (0.02)
Normal bone mass	1.65 (0.01)	1.65 (0.03)	1.75 (0.02)	1.76 (0.05)
Low bone mass	1.58 (0.02)	1.58 (0.04)	1.74 (0.02)	1.75 (0.03)
Osteoporosis	1.64 (0.09)	1.48 (0.12)	1.65 (0.04)	1.59 (0.07)
**Total daily intake of meats, times/day**				
All participants	0.69 (0.001)	0.67 (0.01)	0.64 (0.004)	0.64 (0.01)
Normal bone mass	0.70 (0.005)	0.67 (0.01)	0.68 (0.01)	0.65 (0.01)
Low bone mass	0.67 (0.01)	0.66 (0.01)	0.63 (0.01)	0.64 (0.01)
Osteoporosis	0.65 (0.03)	0.64 (0.05)	0.60 (0.01)	0.61 (0.03)
**Total daily intake of whole grains, times/day**				
All participants	0.74 (0.01)	0.75 (0.01)	0.65 (0.006)	0.67 (0.01)
Normal bone mass	0.73 (0.01)	0.76 (0.02)	0.63 (0.01)	0.66 (0.02)
Low bone mass	0.77 (0.01)	0.74 (0.02)	0.65 (0.01)	0.66 (0.02)
Osteoporosis	0.73 (0.05)	0.59 (0.08)	0.64 (0.02)	0.70 (0.04)
**Total daily intake of calcium fortified foods and beverages, times/day**				
All participants	0.13 (0.004)	0.11 (0.01)	0.18 (0.005)	0.17 (0.01)
Normal bone mass	0.12 (0.005)	0.10 (0.01)	0.16 (0.01)	0.15 (0.01)
Low bone mass	0.14 (0.01)	0.11 (0.01)	0.19 (0.01)	0.17 (0.01)
Osteoporosis	0.21 (0.03)	0.18 (0.06)	0.24 (0.02)	0.19 (0.03)
**Total daily intake of fibre, times/day**				
All participants	0.42 (0.01)	0.40 (0.01)	0.38 (0.01)	0.37 (0.01)
Normal bone mass	0.42 (0.01)	0.41 (0.01)	0.36 (0.01)	0.36 (0.02)
Low bone mass	0.43 (0.01)	0.37 (0.02)	0.40 (0.01)	0.37 (0.01)
Osteoporosis	0.49 (0.04)	0.53 (0.06)	0.40 (0.02)	0.40 (0.04)

BMI, body mass index; PASE, Physical Activity Scale for the Elderly. Data are presented as mean (SE).

^a^ Real personal income and household income was calculated using the month/year of data collection, along with the province of residence in terms of the CPI reference year 2002 (2002 = 100).

### 3.2 Having fewer than 20 teeth was not associated with hip BMD in the combined sample

In the linear regressions using the combined study population – that is, respondents not separated into one of three categories: normal bone mass, low bone mass, or osteoporosis – we did not find a statistically significant association between having fewer than 20 teeth and hip BMD [Model 1 (t = −0.010, *P* = 0.992), Model 2 (t = 0.491, *P* = 0.623), and Model 3 (t = 1.117, *P* = 0.264)] (Table 3). However, each of these models (1,2,3) had some explanatory power [Model 1 (F = 402.591, *P* < 0.001), Model 2 (F = 1,293.873, *P* < 0.001), and Model 3 (F = 423.315, *P* < 0.001)]. For example, Model 3 demonstrated that females were more likely to have a lower hip BMD than males (*P* < 0.001); current smokers had significantly lower hip BMD than non-smokers (*P* < 0.001); and “other” ethnicities had significantly lower hip BMD than “White” ethnicity (*P* = 0.008). Additionally, hip BMD decreased with aging (*P* < 0.001); whereas for every unit increase in BMI (*P* < 0.001), total household income (*P* < 0.001), PASE score (*P* < 0.001), and daily intake of fruits (*P* = 0.043), dairy (*P* < 0.001), whole grains (*P* = 0.048), and fiber (*P* < 0.001), hip BMD increased (Table 3). In contrast, total personal income, and daily intake of vegetables (*P* = 0.805), legumes (*P* = 0.627), nuts (*P* = 0.190), fish (*P* = 0.448), meats (*P* = 0.995), and calcium-fortified products (*P* = 0.119) were not statistically significant in the model.

**Table 3:** Combined study population (*n* = 20,629): using fewer than 20 or more teeth to predict hip BMD when adjusted for demographic (age, sex, ethnicity, BMI, real personal and household income) and lifestyle habits (current smoking status, physical activity level, diet).

Model^a^		Unstandardized coefficients		Standardized coefficients	t	Sig.
		B	Std. error	Beta^b^		
Model 1	(Constant)	0.940	0.006		144.806	<0.001
	Fewer than 20 teeth	−2.254E-05	0.002	−6.899E-05	−0.010	0.992
	Age, years	−0.003	1.009E-04	−0.194	−27.016	<0.001
Model 2	(Constant)	0.767	0.007		103.329	<0.001
	Fewer than 20 teeth	0.001	0.003	0.004	0.491	0.623
	Age, years	−0.003	8.880E-05	−0.195	−30.847	<0.001
	Male	−0.094	0.002	−0.364	−54.931	<0.001
	Ethnicity	0.003	0.002	0.011	1.775	0.076
	BMI, kg/m^2^	0.007	1.422E-04	0.304	50.961	<0.001
	Non-smoker	0.017	0.003	0.038	6.294	<0.001
Model 3	(Constant)	0.705	0.009		76.306	<.001
	Fewer than 20 teeth	0.002	0.002	0.007	1.117	0.264
	Age, years	−0.002	8.880E-05	−0.171	−24.082	<0.001
	Male	−0.092	0.002	−0.356	−54.771	<0.001
	Ethnicity	0.004	0.002	0.016	2.652	0.008
	BMI, kg/m^2^	0.007	1.442E-04	0.314	51.861	<0.001
	Non-smoker	0.013	0.003	0.030	4.887	<0.001
	Total personal income^c^	1.721e-8	3.691E-08	0.004	0.466	0.641
	Total household income^c^	1.915e-7	3.197E-08	0.052	5.991	<0.001
	PASE Score	6.013e-5	1.150E-05	0.034	5.228	<0.001
	Total daily intake of fruits	0.002	0.001	0.013	2.021	0.043
	Total daily intake of vegetables	0.000	0.001	−0.002	−0.248	0.805
	Total daily intake of legumes	−0.001	0.003	−0.003	−0.485	0.627
	Total daily intake of nuts	0.002	0.002	0.008	1.311	0.190
	Total daily intake of fish	−0.003	0.004	−0.005	−0.760	0.448
	Total daily intake of dairy	0.005	0.001	0.040	6.425	<0.001
	Total daily intake of meats	−6.860e-8	1.229E-06	−3.298E-04	−0.056	0.955
	Total daily intake of whole grains	0.003	0.001	0.012	1.978	0.048
	Total daily intake of calcium-fortified products	−0.003	0.002	−0.010	−1.561	0.119
	Total daily intake of fibre	0.005	0.002	0.021	3.341	<0.001

BMD, bone mineral density; BMI, body mass index; PASE, Physical Activity Scale for the Elderly.

^a^ Dependent variable: hip BMD.

^b^ Every 1 unit increase of the continuous variable, or the reference outcome for the categorical variable, changes the odds of having fewer than 20 natural teeth by a factor equivalent to the Beta value (value > 1: positive association between the predictor variable and hip BMD; value < 1: negative association between the predictor variable and hip BMD; value = 1: no association between the predictor variable and hip BMD).

^c^ Real personal income and real household income were calculated using the month/year of data collection, along with the province of residence in terms of the CPI reference year 2002 (2002 = 100).

### 3.3 Having fewer than 20 teeth is associated with lower hip BMD in individuals categorized as having low bone mass but not in individuals with normal bone mass or osteoporosis

Given the range of BMD values in the entire sample, we re-estimated Model 3 separately on each of three inclusive groups based upon each individual's BMD status. This was categorized using the T-score classification of normal bone mass, low bone mass or osteoporosis. Results are shown in Table 4.

**Table 4:** Categorization of population by hip BMD T-score (normal bone mass, *n* = 11,522; low bone mass, *n* = 7,994; or osteoporosis, *n* = 1,113): using fewer than 20 teeth to predict hip BMD when adjusted for demographic (age, sex, ethnicity, BMI, real personal and household income) and lifestyle habits (current smoking status, physical activity level, diet).

Model 3^a^		Unstandardized coefficients		Standardized coefficients	t	Sig.
		B	Std. Error	Beta^b^		
Normal bone mass	(Constant)	0.790	0.010		76.262	<0.001
	Fewer than 20 teeth	0.008	0.002	0.033	3.446	<0.001
	Age, years	−0.001	1.131E-04	−0.094	−8.809	<0.001
	Male	−0.045	0.002	−0.224	−22.858	<0.001
	Ethnicity	0.008	0.002	0.039	4.406	<0.001
	BMI, kg/m^2^	0.004	1.573E-04	0.224	24.626	<0.001
	Non-smoker	0.007	0.003	0.020	2.200	0.028
	Total personal income^c^	−2.680e-8	3.963E-08	−0.009	−0.676	0.499
	Total household income^c^	1.102e-7	3.539E-08	0.041	3.114	0.002
	PASE Score	9.338e-6	1.212E-05	0.007	0.770	0.441
	Total daily intake of fruits	0.002	0.001	0.020	2.023	0.043
	Total daily intake of vegetables	−0.001	0.001	−0.006	−0.575	0.565
	Total daily intake of legumes	0.001	0.003	0.004	0.470	0.638
	Total daily intake of nuts	0.003	0.002	0.014	1.481	0.139
	Total daily intake of fish	0.003	0.005	0.004	0.488	0.625
	Total daily intake of dairy	0.004	0.001	0.042	4.514	<0.001
	Total daily intake of meats	1.641e-7	1.015E-06	0.001	0.162	0.872
	Total daily intake of whole grains	0.001	0.002	0.006	0.654	0.513
	Total daily intake of calcium-fortified products	−0.002	0.002	−0.007	−0.727	0.467
	Total daily intake of fibre	0.001	0.002	0.005	0.494	0.621
Low bone mass	(Constant)	0.655	0.006		117.423	<0.001
	Fewer than 20 teeth	−0.002	0.001	−0.024	−2.058	0.040
	Age, years	−0.001	6.063E-05	−0.109	−8.458	<0.001
	Male	−0.016	0.001	−0.175	−15.014	<0.001
	Ethnicity	−8.493e-6	0.001	−9.213E-05	−0.008	0.993
	BMI, kg/m^2^	0.002	9.857E-05	0.188	16.916	<0.001
	Non-smoker	0.003	0.002	0.021	1.859	0.063
	Total personal income^c^	3.487e-8	2.294E-08	0.022	1.520	0.129
	Total household income^c^	8.070e-9	1.919E-08	0.006	0.421	0.674
	PASE Score	2.218e-5	7.366E-06	0.036	3.011	0.003
	Total daily intake of fruits	−1.896E-04	0.001	−0.004	−0.359	0.719
	Total daily intake of vegetables	1.600E-04	0.001	0.004	0.307	0.758
	Total daily intake of legumes	−0.002	0.002	−0.013	−1.138	0.255
	Total daily intake of nuts	4.213E-04	0.001	0.005	0.469	0.639
	Total daily intake of fish	−0.002	0.003	−0.010	−0.874	0.382
	Total daily intake of dairy	0.001	4.588E-04	0.014	1.271	0.204
	Total daily intake of meats	1.510E-04	0.001	0.001	0.111	0.912
	Total daily intake of whole grains	0.003	0.001	0.034	3.126	0.002
	Total daily intake of calcium-fortified products	−1.813E-04	0.001	−0.002	−0.162	0.871
	Total daily intake of fibre	0.001	0.001	0.012	1.035	0.301
Osteoporosis	(Constant)	0.524	0.012		45.418	<0.001
	Fewer than 20 teeth	−0.002	0.002	−0.029	−0.915	0.360
	Age	−2.281E-04	1.275E-04	−0.060	−1.788	0.074
	Male	−0.001	0.003	−0.007	−0.225	0.822
	Ethnicity	−0.001	0.002	−0.017	−0.551	0.582
	BMI, kg/m^2^	0.001	2.206E-04	0.135	4.403	<0.001
	Non-smoker	0.002	0.004	0.018	0.594	0.553
	Total personal income^c^	7.827e-8	5.770E-08	0.057	1.356	0.175
	Total household income^c^	5.023e-9	4.417E-08	0.005	0.114	0.909
	PASE Score	5.147e-5	1.731E-06	0.094	2.974	0.003
	Total daily intake of fruits	−3.804E-04	0.001	−0.011	−0.336	0.737
	Total daily intake of vegetables	0.003	0.001	0.079	2.396	0.017
	Total daily intake of legumes	0.001	0.003	0.006	0.198	0.843
	Total daily intake of nuts	−0.001	0.002	−0.016	−0.525	0.600
	Total daily intake of fish	−0.008	0.006	−0.045	−1.472	0.141
	Total daily intake of dairy	0.001	0.001	0.016	0.533	0.594
	Total daily intake of meats	−0.003	0.003	−0.033	−1.077	0.282
	Total daily intake of whole grains	0.001	0.002	0.009	0.299	0.765
	Total daily intake of calcium-fortified products	−0.002	0.002	−0.028	−0.894	0.372
	Total daily intake of fibre	−0.006	0.002	−0.088	−2.889	0.004

BMD, bone mineral density; BMI, body mass index; PASE, Physical Activity Scale for the Elderly.

^a^ Dependent variable: hip BMD.

^b^ Every 1 unit increase of the continuous variable, or the reference outcome for the categorical variable, changes the odds of having fewer than 20 teeth by a factor equivalent to the Beta value (value > 1: positive association between the predictor variable and hip BMD; value < 1: negative association between the predictor variable and hip BMD; value = 1: no association between the predictor variable and hip BMD).

^c^ Real personal income and real household income were calculated using the month/year of data collection, along with the province of residence in terms of the CPI reference year 2002 (2002 = 100).

Individuals with normal bone mass: Lower hip BMD was associated with having 20 or more teeth (*P* < 0.001). In addition, higher BMD was positively associated with being female (*P* < 0.001), a non-smoker (*P* = 0.028), and self-reporting a White ethnicity (*P* < 0.001). Hip BMD decreased with aging (*P* < 0.001) and increased with every unit increase in BMI (*P* < 0.001), total household income (*P* = 0.002), and daily intake of fruits (*P* = 0.043) and dairy (*P* < 0.001).

Individuals with low bone mass: Lower hip BMD was associated with having fewer than 20 teeth (*P* = 0.018). In addition, females had lower hip BMD compared with males (*P* < 0.001) and hip BMD decreased with aging (*P* < 0.001). The model also identified hip BMD as increasing with higher BMI (*P* < 0.001), PASE score (*P* = 0.003), and daily intake of whole grains (*P* = 0.002).

Individuals with osteoporosis: There was no significant association between having 20 or more teeth vs. fewer than 20 teeth among individuals with osteoporosis (*P* = 0.360). Hip BMD increased with higher BMI (*P* < 0.001), PASE score (*P* = 0.003), and greater daily intake of vegetables (*P* = 0.017) and fibre (*P* = 0.004).

## 4 Discussion

There was no statistically significant association between hip BMD and being identified as either having 20 or more natural teeth vs. fewer than 20 natural teeth in the combined sample of Canadian men and women aged 50–85 years. However, given the large range of BMD values observed in this sample, we re-estimated the model using data on three sub-samples. Using each individual's BMD, we categorized the individuals using T-scores into one of three groups: normal bone mass, low bone mass and osteoporosis. By refining the samples, we were able to obtain more precise estimates of the association between having fewer than 20 teeth and BMD. Specifically, for the low bone mass group, having fewer than 20 teeth was associated with lower BMD. This subgroup also had the largest number of women, which was not unexpected given the known decline in BMD with aging. The opposite relationship between hip BMD and number of teeth (<20 vs. ≥ 20 teeth) was seen in individuals with normal bone mass. However, this group also showed the greatest variation in socio-demographic characteristics and was the youngest and most financially secure group. Finding an absence of negative or positive association between fewer than 20 teeth and hip BMD among individuals with osteoporosis was unsurprising for a few reasons. It was the smallest group and the ratio of women to men is 5:1. In addition, individuals in this group may have already been identified as having osteoporosis and therefore potentially used medications to prevent further loss of BMD and fractures. While it would have been preferable to have included information on medication use, this data was incomplete in the sample and could not be incorporated. Further, individuals living with osteoporosis may have had greater motivation to consume foods or use supplements that particularly support bone health. The inconsistent findings between the combined sample and normal bone mass, low bone mass, and osteoporosis subgroups may have been due to the differences in group characteristics (e.g., sex ratio, average income, medication use) but understanding this unexpected relationship warrants further investigation.

Comparing the findings from the present study with published studies is complex as most studies have measured number of teeth as a continuous variable, others have used different skeletal sites (e.g., spine or metacarpal), and the relationship between tooth loss and BMD has been predominantly studied in postmenopausal women. In a recent study in Thailand, postmenopausal women, as well as the pooled results from men and women aged 30–82 years, showed that osteoporosis (described by T-score classification using hip BMD) was associated with increased tooth loss (51). A cross-sectional study, the National Health and Nutrition Examination Survey (NHANES 2005–2006, 2007–2008, 2009–2010, 2013–2014, and 2017–2018), inclusive of men and women age 20 years and above, also demonstrated that fewer teeth was associated with a greater likelihood of lower BMD at the femur neck, as well as a reported hip fracture (52). Findings from the Korean National Health and Nutrition Examination Survey (KNHANES 2011–2012) resulted in similar findings in which postmenopausal women aged 50–80 with lower BMD at the femoral neck or lumbar spine had a higher risk of tooth loss, compared with women with “normal” BMD (53) – this was not seen in men, as per the KNHANES 2008–2010 (54). Each of these studies included data in which the number of teeth was measured as a continuous variable. No studies have been conducted to support that the 20 teeth cutoff is associated with systemic bone changes (i.e., lower BMD or higher risk of fracture), with exception to one study investigating the cut-off of 20 teeth in relation to BMD at the metacarpal region. In Japanese postmenopausal women (mean age = 63.3 ± 7.7 years), having fewer than 20 teeth was associated with a greater likelihood of having lower metacarpal BMD, as measured using an x-ray radiograph of the hand standardized to an aluminum step wedge, compared with having more than 20 teeth (32). Hip BMD was not examined (32). In the current study, the absence of an association between number of teeth and hip BMD in the combined sample may be due to the dichotomous measurement of tooth loss. The different findings depending on the categorization of BMD emphasizes the importance of measuring tooth loss as a continuous measure and/or consideration of additional oral health parameters to more clearly identify if there is a relationship between tooth loss and BMD.

As expected, the inclusion of covariates that are major risk factors known to influence bone health (age, sex, ethnicity, BMI, current smoking status, income, and diet) strengthened the model. Tooth loss with aging has been well documented, and the findings from the present study support this association within the age range of 50–85 years. Examining adults who were at least age 50 years was pertinent in order to align with the age cutoffs defined within guidelines used to support bone health, including the Clinical Practice Guideline for Management of Osteoporosis and Fracture Prevention in Canada: 2023 update (3). Being female was also shown to be associated with having lower hip BMD in the combined sample, as well as in the low bone mass group. This aligned with data showing a greater risk of osteoporosis in females compared with males (3). However, among individuals with normal bone mass, being female was associated with having higher BMD. This could be because these females may not have the typical risk factors for osteoporosis. Moreover, this group included more males (2:1 male to female ratio). The lack of difference between males and females in the osteoporosis group may have been due to the smaller sample size and/or potential medication use that is stabilizing the loss of BMD.

The average retirement age in Canada is 65.1 years (55); and during the transition out of the labour force, there is a loss of employment health benefits, as well as the likelihood of a reduction in income (56). The analysis of the combined sample showed that higher total household income was associated with higher hip BMD, though this association was not present in the low bone mass or osteoporosis groups. The positive association in the combined analysis was likely due to the fact that income impacts lifestyle habits that affect an individual's health, including food choices. In 2021, food insecurity due to financial constraints affected 18.4% of Canadian households (57), and with higher food prices, this number continues to rise. Of concern is the prevalence of micronutrient inadequacies (calcium, magnesium, vitamin D, vitamin B6, zinc, and folate) that have been observed among any severity of food insecurity, with the greatest prevalence demonstrated in severe food insecure households (58). There is also an inverse association between diet quality and tooth loss, in which adults with more severe tooth loss were less likely to consume an adequate diet (59). Moreover, compromised dentition and/or an inability to chew foods is associated with a greater likelihood of nutrient deficiencies (60). Consuming a balanced diet – defined as a diet that aligns with Canada's Food Guide (Canada.ca/FoodGuide) – that includes bone promoting nutrients plays a key role in the prevention of osteoporosis and related fractures during aging (3). Canada's Food Guide emphasizes the consumption of vegetables and fruits, whole grains, and protein foods that include dairy. Within each analysis there were some significant associations that align with this dietary guidance. In the analysis of the combined sample, this was shown through the association of a higher intake of dairy, fruits, whole grains and fibre with higher hip BMD. The food groups included in the current study analyses were selected *a priori* based on their source of bone supporting nutrients. While not all food categories were associated with hip BMD, it is acknowledged that the differences in the occurrence of daily food group intakes were minimal. Understanding the relationship between tooth retention, adequate diet, and skeletal BMD is important for determining diet strategies to support overall bone health and is an area for future study.

Along with diet, physical activity is a modifiable risk factor with beneficial health effects, including prevention for osteoporosis (3, 7). Mean PASE scores of the participants were similar to those previously reported among community-dwelling Canadian older adults approximately around ages 50–79 years (61). A higher PASE score was associated with higher hip BMD in the combined sample, as well as, in the low bone mass and osteoporosis subgroups. The reason why there was no significant relationship in PASE score in the normal bone mass group is unclear. Muscle-strengthening exercises have beneficial effects on BMD, while incorporating balance activities has been encouraged to reduce the risk of falls (3). Though there has been a lack of correlation demonstrated between PASE scores and BMI (62), adequate diet and physical activity play a key role in improving BMI towards maintaining a healthy weight. The average BMI of men and women in this present study was classified as overweight, and increasing BMI was associated with a greater hip BMD in each analysis. Numerous studies have reported a positive association between BMI and BMD, as described by a U-shaped relationship whereby extremely underweight or overweight individuals have an increased risk of fracture (63). Tomlinson et al. attributed greater BMD in older adults with high BMI to a combination of greater mechanical loading and greater total caloric intake (64). Ethnicity was also considered as a covariate given discussion of potential ethnic differences in bone health (65). Of note is that the sample population was 97.1% self-reported White ethnicity and this high percentage may have influenced the finding of White ethnicity being significantly associated with hip BMD in the combined sample as well as the subgroup categorized with normal BMD.

### 4.1 Limitations

A major strength of this study was the availability of many variables, which allowed for consideration of individual characteristics as covariates within this large study population. Limitations include the dichotomous measurement of number of teeth, the sample population and the cross-sectional design of the present study. Examining the number of teeth as a dichotomous rather than a continuous variable limited the variation in the dependent variable, such that there were only two possible outcomes that were observed at a single timepoint. Of note, a total of 81% of the participants within the present study (*n* = 16, 746) reported having 20 or more natural teeth. In addition, the sample population was not representative of the Canadian demographic as recruitment was localized to urban regions in proximity to a data collection site (43). This resulted in a population that was largely comprised of healthy, well-educated individuals with a total household income that is higher than the Canadian median. Also, the use of cross-sectional data means that no causal relationship between hip BMD and the predictor variables could be defined.

## 5 Conclusion

This study demonstrated that having fewer than 20 natural teeth was not significantly associated with having a lower hip BMD in the combined sample of Canadian men and women who were between 50 and 85 years of age. However, upon classifying each individual into a sub-sample based upon bone health status - according to hip BMD - the results changed. For individuals with low BMD there was a statistically significant relationship between having fewer than 20 teeth and having a lower hip BMD. Of note is that these individuals may be most at risk for osteoporosis and, perhaps, less aware of their bone health status. The significant association between demographic (e.g., age, sex, BMI) or lifestyle variables (e.g., diet and physical activity) and BMD suggests the importance of considering these covariates when planning future studies. Moreover, whether including other indicators of oral health - in addition to number of teeth - can strengthen the ability to predict hip BMD with or without considering hip BMD status is an area of future study.

## Data Availability

The datasets presented in this article are not readily available because an application to access the de-identified data is required. Requests to access the datasets should be directed to Canadian Longitudinal Study on Aging, access@clsa-elcv.ca.
